# Effect of TDA‐producing *Phaeobacter inhibens* on the fish pathogen *Vibrio anguillarum* in non‐axenic algae and copepod systems

**DOI:** 10.1111/1751-7915.13275

**Published:** 2018-05-06

**Authors:** Bastian Barker Rasmussen, Katrine Ege Erner, Mikkel Bentzon‐Tilia, Lone Gram

**Affiliations:** ^1^ Department of Biotechnology and Biomedicine Technical University of Denmark Anker Engelundsvej bldg. 301 DK‐2800 Kgs. Lyngby Denmark

## Abstract

The expanding aquaculture industry plays an important role in feeding the growing human population and with the expansion, sustainable bacterial disease control, such as probiotics, becomes increasingly important. Tropodithietic acid (TDA)‐producing *Phaeobacter* spp. can protect live feed, for example rotifers and *Artemia* as well as larvae of turbot and cod against pathogenic vibrios. Here, we show that the emerging live feed, copepods, is unaffected by colonization of the fish pathogen *Vibrio anguillarum*, making them potential infection vectors. However, TDA‐producing *Phaeobacter inhibens* was able to significantly inhibit *V. anguillarum* in non‐axenic cultures of copepod *Acartia tonsa* and the copepod feed *Rhodomonas salina*. *Vibrio* grew to 10^6^ CFU ml^−1^ and 10^7^ CFU ml^−1^ in copepod and *R. salina* cultures, respectively. However, vibrio counts remained at the inoculum level (10^4^ CFU ml^−1^) when *P. inhibens* was also added. We further developed a semi‐strain‐specific qPCR for *V. anguillarum* to detect and quantify the pathogen in non‐axenic systems. In conclusion, *P. inhibens* efficiently inhibits the fish larval pathogen *V. anguillarum* in the emerging live feed, copepods, supporting its use as a probiotic in aquaculture. Furthermore, qPCR provides an effective method for detecting vibrio pathogens in complex non‐axenic live feed systems.

## Introduction

Aquaculture provides high‐quality protein for approximately three billion people worldwide and with the estimated increase in the human population combined with overfishing, this number is likely to increase (WWF and ZSL, 2015; FAO, 2016). Larvae of several commercially important species including cod, halibut and turbot are fed using live feed due to the lack of suited artificial feed formulations. Traditionally, marine fish larvae have been fed using rotifers and *Artemia,* although copepods are more representative of the natural diet (Turner, 2004). Copepods have several advantages over traditional aquaculture live feed including desirable amino acid profiles, a high fatty acid content and swimming patterns that promote feeding in fish larvae (Støttrup *et al*., 1986; Støttrup and Norsker, 1997; Bell *et al*., 2003; Turingan *et al*., 2007). Furthermore, substituting rotifers with copepods (*Acartia tonsa*) in the initial larval feeding stage has a long‐term positive effect on survival, growth and viability of Atlantic cod and ballan wrasse fish larvae (Øie *et al*., 2017). So far, the use of copepods as live feed in larval rearing has not been cost‐effective; however, new approaches to copepod breeding are improving the cost balance (Abate *et al*., 2016), thus making copepods a relevant live feed in commercial aquaculture.

A major bottleneck in aquaculture is disease outbreaks where bacterial infections account for approx. 55% (Kibenge *et al*., 2012). One of the most prominent bacterial genera causing disease in aquaculture is *Vibrio* including *Vibrio anguillarum, V. harveyi, V. vulnificus* and *V. splendidus* (Thompson *et al*., 2004; Toranzo *et al*., 2005). Live feed used in aquaculture is a potential point of entry and vector for pathogenic bacteria (Olafsen, 2001), and several *Vibrio* species, including the human and fish pathogens *V. cholerae, V. vulnificus* and *V. splendidus,* are found in association with zooplankton, for example copepods, which in the marine environment seem to serve as natural *Vibrio* reservoirs (Sochard *et al*., 1979; Heidelberg *et al*., 2002; Colwell *et al*., 2003; Vezzulli *et al*., 2015). The role of copepods as hosts for *V. cholerae* has especially been studied extensively, and *V. cholerae* has repeatedly been isolated from the surface, gut and exuviae of these small crustaceans (Huq *et al*., 1983; Tamplin *et al*., 1990; Gugliandolo *et al*., 2008). Thus, although vibrios such as *Vibrio parahaemolyticus*,* V. alginolyticus* and *V. mimicus* have been reported to be unable to colonize the copepod species *Temora stylifera*,* Acartia clausi*,* Centropages typicus* and *Paracalanus parvus* (Dumontet *et al*., 1996), it is a valid concern that *Acartia tonsa* may function as infection vectors for other *Vibrio* species including the fish pathogenic *V. anguillarum* in aquaculture systems.

Historically, antibiotics have been used to control bacterial infections in aquaculture. However, risk of antibiotic resistance development and the associated health risk have led to a search for sustainable alternatives (Skjermo and Vadstein, 1999; Sommerset *et al*., 2005; Defoirdt *et al*., 2011). The deployment of vaccines has limited the use of antibiotics especially in Europe and North America (Defoirdt *et al*., 2011; Ringø *et al*., 2014). However, antibiotics are still used in many countries and fish larviculture as their undeveloped immune system does not allow for vaccination (Sommerset *et al*., 2005; Defoirdt *et al*., 2011). An alternative to antibiotics in such systems is probiotics, that is live microorganisms which exert beneficial effects on the host health (FAO and WHO, 2001), for example by inhibiting growth of pathogenic bacteria (Gatesoupe, 1999; Skjermo and Vadstein, 1999; Kesarcodi‐Watson *et al*., 2008). Members of the genera *Phaeobacter* and *Ruegeria* which produce the broad‐spectrum antimicrobial compound tropodithietic acid (TDA) have proven probiotic properties (Ruiz‐Ponte *et al*., 1999; Hjelm *et al*., 2004; Planas *et al*., 2006; D'Alvise *et al*., 2012). *Phaeobacter* spp. inhibit the growth of pathogenic vibrios and protect live feed such as rotifers and *Artemia* (D'Alvise *et al*., 2012; Grotkjær *et al*., 2016a,b). Furthermore, *Phaeobacter* spp. can protect larvae of turbot and cod in challenge trials (Planas *et al*., 2006; D'Alvise *et al*., 2012, 2013). *Phaeobacter* spp. have repeatedly been isolated from different aquaculture facilities (Ruiz‐Ponte *et al*., 1998; Hjelm *et al*., 2004; Porsby *et al*., 2008; Gram *et al*., 2015; Grotkjær *et al*., 2016b), which indicate that they are part of the natural microbiota in these aquaculture units. Most studies on the probiotic effect of *Phaeobacter* spp. and *Ruegeria* spp. have been carried out in axenic live feed or fish larvae systems (Planas *et al*., 2006; D'Alvise *et al*., 2012, 2013; Grotkjær *et al*., 2016b). Although non‐axenic algae and *Artemia* systems have been tested (Grotkjær *et al*., 2016a), the probiotic effect of *Phaeobacter* spp. and *Ruegeria* spp. in these more ‘natural’ systems requires further studies.

The purpose of this study was to investigate whether the copepod species *Acartia tonsa* would potentially function as an infection vector for fish pathogenic vibrio, and whether the TDA‐producing *P. inhibens* could antagonize *V. anguillarum* in non‐axenic copepod systems as well as in algae used as copepod feed. Furthermore, we aimed at developing a strain‐specific quantitative PCR (qPCR) protocol for detection and quantification of pathogens in complex systems without the dependency on selection markers, such as antibiotic resistance that potentially could affect pathogen behaviour.

## Results

### Invasion of copepods by GFP‐tagged *V. anguillarum*

**Figure 1:**
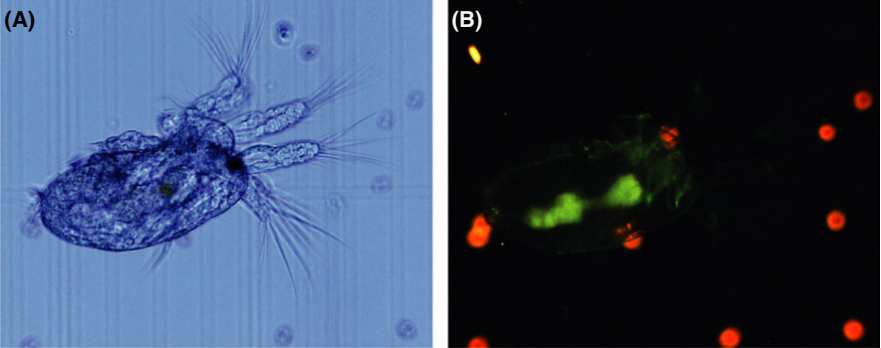
Microscopy of *A. tonsa* nauplii colonized by GFP‐tagged *V. anguillarum* NB10. Red fluorescent cells are *R. salina*. Magnification 100 ×  A. Phase‐contrast microscopy. B. Fluorescence microscopy (WIB excitation 460–490 nm, emission > 515 nm).

To test whether *V. anguillarum* could invade the copepod *Acartia tonsa* and make the crustacean a potential infection vector in larviculture, a GFP‐tagged *V*. *anguillarum* strain NB10 was added to non‐axenic *A. tonsa* feed, that is *Rhodomonas salina*. After 4 days of incubation, the GFP signal of NB10 was detected on the surface and inside the copepods (Fig. 1). Specifically, the vibrios appeared to be concentrated within the intestinal system of the crustacean. No GFP signal was detected in non‐inoculated copepods. Throughout the experiment, *Vibrio* abundances in the surrounding medium were stable around the inoculum level of 10^5^ CFU ml^−1^ (data not shown). Furthermore, *A. tonsa* seemed unaffected by the addition of vibrios, exhibiting mortalities after 4 days of 1.47 ± 0.72%.

### Co‐culture in non‐axenic *Rhodomonas salina*

**Figure 2:**
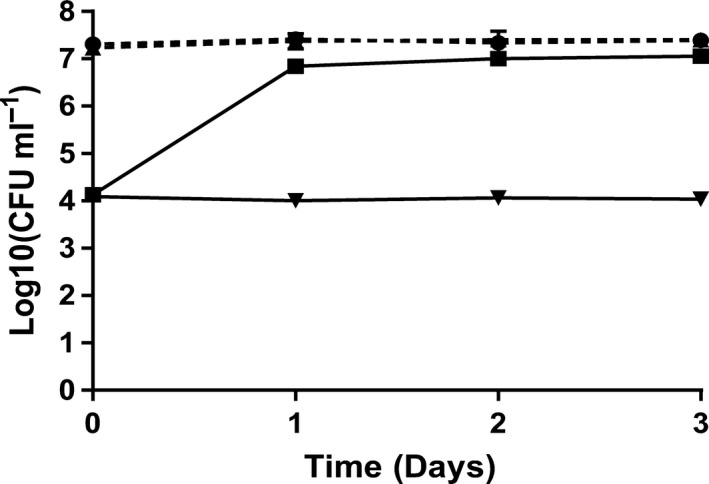
Colony counts of *V. anguillarum* 90‐11‐286 and *P. inhibens* DSM 17395 during a co‐culture experiment in non‐axenic *R. salina* cultures. *V. anguillarum* without *P. inhibens* (■, solid line), *V. anguillarum* with *P. inhibens* (▼, solid line), *P. inhibens* without *V. anguillarum* (●, dashed line) and *P. inhibens* with *V. anguillarum* (▲, dashed line). The two *P. inhibens* lines are situated on top of one another. Error bars show the standard deviation of three biological replicates.

As *A. tonsa* was found to be a potential vector for *V*. *anguillarum,* we investigated whether *P. inhibens* could antagonize vibrios in cultures of copepod and in the copepod live feed *R. salina*. We conducted a co‐culture experiment in non‐axenic *R. salina* cultures. Counts of *R. salina* were stable during the co‐culture experiment, and no significant difference was seen between the differently treated cultures (data not shown). *Phaeobacter inhibens* established itself and remained at approx. 10^7^ CFU ml^−1^ in all set‐ups, independently of addition of *V. anguillarum* strain 90‐11‐286 (Fig. 2). TSA counts showed that *V. anguillarum* was significantly reduced when grown in the presence of *P. inhibens*. Without *P. inhibens*,* V. anguillarum* grew to approx. 10^7^ CFU ml^−1^, while in the presence of *P. inhibens*,* V. anguillarum* numbers were significantly reduced with approximately three orders of magnitude (*P *<* *0.0001) and never exceeded the inoculum level of 10^4* *^CFU ml^−1^.

### Co‐culture in non‐axenic *A. tonsa,* and detection and quantification of *V. anguillarum* with qPCR

**Figure 3:**
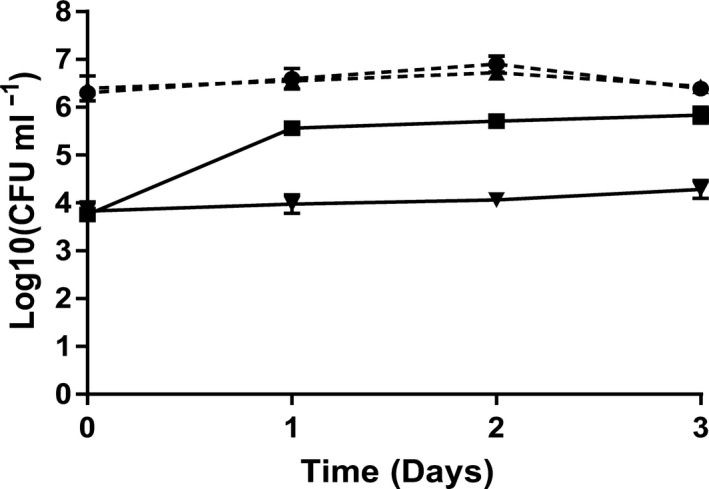
Colony counts of *V. anguillarum* 90‐11‐286 and *P. inhibens* DSM 17395 during co‐culture in non‐axenic *A. tonsa* cultures. *V. anguillarum* without *P. inhibens* (■, solid line), *V. anguillarum* with *P. inhibens* (▼, solid line), *P. inhibens* without *V. anguillarum* (●, dashed line) and *P. inhibens* with *V. anguillarum* (▲, dashed line). Error bars show the standard deviation of three biological replicates.

Non‐axenic *A. tonsa* were not affected by the presence of either the probiont or the pathogen resulting in mortalities after 3 days of 5.7 ± 2.52%, 7.3 ± 1.15%, 5.7 ± 2.08% and 6.3 ± 3.51% in cultures with *P. inhibens*,* V. anguillarum*, a combination of *P. inhibens* and *V. anguillarum*, or no addition (control), respectively. As for the *R. salina* co‐culture experiment, no differences were observed between *P. inhibens* abundances in the presence or absence of *V. anguillarum* (Fig. 3) with counts being stable around the inoculum level of 10^6^–10^7^ CFU ml^−1^. The abundance of *V. anguillarum* was significantly reduced by *P. inhibens* (*P *=* *0.0004). With the addition of *P. inhibens*,* Vibrio* counts remained at the inoculum level around 10^4^ CFU ml^−1^, approx. two orders of magnitude below the abundances observed in cultures without *P. inhibens*. Numbers of *V. anguillarum* were estimated both by counts on TSA plates and by qPCR resulting in similar abundances (*P *>* *0.05; Table 1).

**Table 1:** Quantification of *V. anguillarum* strain 90‐11‐286 using colony counts and qPCR. Values are presented as log10‐transformed counts. Standard deviations are based on three biological replicates. *P* values were calculated using *t*‐test (alpha = 0.05) and describe differences between TSA plate counts and *C*_*t*_ based counts

Time (days)	*V. anguillarum* counts without *P. inhibens*			*V. anguillarum* counts with *P. inhibens*		
	TSA	*C*_*t*_ based	*P* value	TSA	*C*_*t*_ based	*P* value
0	3.76 ± 0.10	3.82 ± 0.14	0.6114	3.83 ± 0.20	3.70 ± 0.16	0.4416
1	5.57 ± 0.11	5.70 ± 0.08	0.1657	3.98 ± 0.20	3.93 ± 0.04	0.7095
2	5.71 ± 0.13	5.72 ± 0.12	0.9621	4.06 ± 0.03	3.83 ± 0.18	0.1016
3	5.84 ± 0.17	5.88 ± 0.17	0.7626	4.28 ± 0.18	3.85 ± 0.21	0.0561

### Verification of primer specificity and standard curve

**Figure 4:**
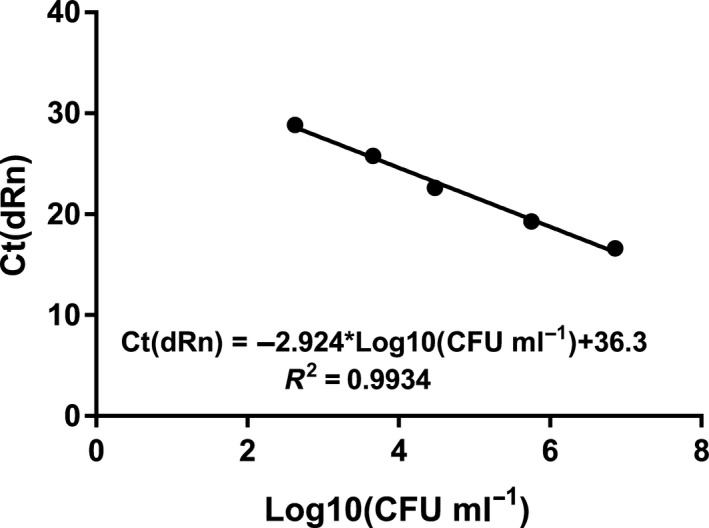
qPCR standard curves for *V. anguillarum* 90‐11‐286 in non‐axenic copepod cultures. Standard curve was obtained by running, in triplicates, DNA extracted from one sample.

Primer‐BLAST was used to design and test the specificity of the *V. anguillarum* 90‐11‐286 primers *in silico,* and this indicated that the primers were strain‐specific. The primers were subsequently tested *in vitro* against 11 *V. anguillarum* strains and one *Vibrio harveyi* strain (Table 2). The primers amplified the DNA of three strains resulting in cycle threshold (*C*_*t*_) values within the limits of detection (*C*_*t*_ ≤ 30). Furthermore, one strain, NB10, was close to the limit of detection with a *C*_*t*_ value of 30.29 ± 0.13. Strains 90‐11‐286 and PF7 showed similar *C*_*t*_ values of 19.56 ± 0.05 and 19.16 ± 0.11, respectively, and a higher *C*_*t*_ value of 23.53 ± 0.08 was seen for PF4. For quantification, a standard curve was made with strain 90‐11‐286 overnight culture that was 10‐fold serial diluted from approx. 10^7^ to 10^3^ (Fig. 4). The standard curve was used to compare *C*_*t*_ values to bacterial density (CFU ml^−1^) for *Vibrio* samples from the non‐axenic *A. tonsa* co‐culture.

**Table 2:** *Vibrio* strains used to test the specificity of the *V. anguillarum* strain 90‐11‐286 primers. Standard deviations are based on technical duplicates

Strain	Species	Isolation		Serotype	Plasmid pJM1	Accession no.		Mean *C*_*t*_ ‐value
		Host	Country			Genome	Plasmid	
4299	*V. anguillarum*	Unknown	Norway	O2b	−	CP011458/CP011459		35.57 ± 1.51
90‐11‐286		Rainbow trout	Denmark	O1	−	CP011460/CP011461		19.56 ± 0.05
90‐11‐287		Rainbow trout	Denmark	O1	+	CP011475/CP011476	CP016254	32.31 ± 0.02
775		Coho salmon	Norway	O1	+	CP002284/CP002285	AY312585	33.41 ± 0.78
H610		Atlantic cod	USA	O2a	−	CP011462/CP011463		37.06 ± 2.11
NB10		Unknown	Sweden	O1	+	LK021130/LK021129	LK021128	30.29 ± 0.13
PF4		Atlantic salmon	Chile	O3	−	CP010080/CP010081		23.53 ± 0.08
PF7		Atlantic salmon	Chile	O3	−	CP011464/CP011465		19.16 ± 0.11
VIB243		Sockeye salmon	USA	O1/VaNT1	+	CP010030/CP010031	CP016261	35.18 ± 0.30
DSM21597		Atlantic cod	Norway	O2	−	CP010084/CP010085		34.31 ± 0.76
S2 2/9		Rainbow trout	Denmark	O1	−	CP011472/CP011473		36.61 ± 2.17
DSM19623	*V. harveyi*	Amphipod	USA	–	−	BAOD01000001		39.58

## Discussion

Live feed is essential at the larval stage in several commercially important species of finfish, and controlling pathogenic bacteria in the live feed is important as these may act as vectors for the pathogens. Here, we demonstrate that *P. inhibens* can inhibit the highly virulent *V. anguillarum* strain 90‐11‐286 in two different non‐axenic live feed systems. Specifically, we demonstrate the probiotic potential in copepods as these are superior as larval feed compared to *Artemia* and rotifers, and future aquaculture is expected to rely increasingly on a copepod‐based live feed.

Live feed is known as a point of entry and vector for pathogenic bacteria (Olafsen, 2001). Copepods have previously been shown to host some *Vibrio* spp. (Huq *et al*., 1983; Tamplin *et al*., 1990; Heidelberg *et al*., 2002; Gugliandolo *et al*., 2008; Vezzulli *et al*., 2015); however, some *Vibrio* spp. seem to be unable to colonize some copepod species (Dumontet *et al*., 1996). Using a GFP‐tagged *V. anguillarum* NB10, we found that *V. anguillarum* was able to colonize the outside and the gut of the feeding copepods without inducing notable mortality (1.47 ± 0.72% after 4 days) hence making them potential infection vectors in larviculture.

*Vibrio* spp. are part of the established microbiota of live feed used in larviculture (Berland *et al*., 1970; Austin and Allen, 1982; Salvesen *et al*., 2000; López‐Torres and Lizárraga‐Partida, 2001) and when introduced, these opportunistic pathogens can proliferate rapidly leading to great losses (Reid *et al*., 2009). In the co‐culture experiment, no differences were seen in mortality of the unfed copepods regardless of the presence of *P. inhibens* or *V. anguillarum* (5.7 ± 2.52% to 7.3 ± 1.15% mortality). In contrast, *V. anguillarum* has been shown to cause high mortality (92%) in non‐axenic *Artemia* cultures. Here, the addition of *P. inhibens* reduced mortality of the infected *Artemia* to 11% (Grotkjær *et al*., 2016b). The observed differences in mortality potentially give copepods an advantage over *Artemia* as live feed, as it has been reported that homogenized *Artemia* or sea bass larval biomass, simulating the presence of dead *Artemia* or sea bass larvae, increase the virulence of *V. anguillarum*, resulting in a significant increase in mortality in challenged sea bass larvae (Li *et al*., 2014). Thus, copepods with a higher survival rate than *Artemia* could result in fewer and potentially less virulent vibrios. The differences in mortality between *Artemia* and copepods can be explained by several factors. A notable difference between the studies is the vibrio abundances observed at the end of the co‐culture experiments. Although inoculated at same level (10^4^ CFU ml^−1^), abundances of *V. anguillarum* in cultures without *P. inhibens* were 10‐fold higher in the *Artemia* experiment (10^7^ CFU ml^−1^) (Grotkjær *et al*., 2016a) as compared to the copepod experiment conducted in the present study (10^6^ CFU ml^−1^). This suggests that the presence of copepods, or the background microbiota of the copepods, could have an inhibiting effect on vibrio growth, especially as vibrio abundances in the algae cultures of both studies, *Tetraselmis suecica* and *R. salina*, respectively, were similar to that of the *Artemia* cultures, that is 10^7^ CFU ml^−1^. The immune systems of invertebrates are poorly understood (Loker *et al*., 2004). However, there are differences between copepods and *Artemia*, and for instance, the heat shock protein *hsp70*, which is associated with pathogen resistance, is induced in *Artemia* when infected with Vibrio (Norouzitallab *et al*., 2016). In contrast, an analog of this stress responder was not induced in the copepod *Eurytemora affinis* when exposed to *Vibrio* spp. (Almada and Tarrant, 2016). Different copepod species are colonized by different bacteria, and some *Vibrio* spp. are unable to colonize some copepod species (Dumontet *et al*., 1996). Vibrios colonizing copepods have been reported to trigger upregulation of saposin‐like genes and C‐type lectins (Almada and Tarrant, 2016) that have antimicrobial properties (Hoeckendorf and Leippe, 2012; Wang *et al*., 2014). This suggests that copepods have a potential inhibiting effect on the growth of *V. anguillarum*, which could explain the lower Vibrio abundance and mortality in copepod cultures relative to *Artemia* cultures. It is also possible that the *V. anguillarum* strain 90‐11‐286 is avirulent against *A. tonsa*. The virulence of strains within the same species can vary immensely as seen for thirty *V. anguillarum* strains tested in three different hosts (turbot, halibut and cod larvae), causing mortality from 100% to 9.1% with the same infection dose (Rønneseth *et al*., 2017). Thus, if other *V. anguillarum* strains had been tested in the *Artemia* and *A. tonsa* systems, the outcome might have been different.

Several co‐culture studies of *P. inhibens* and *V. anguillarum* in axenic systems have shown significant inhibition of vibrios, resulting in lowered vibrio counts relative to the inoculum level (D'Alvise *et al*., 2010, 2012; Grotkjær *et al*., 2016b). In contrast, *P. inhibens* kept *V. anguillarum* at the inoculum level in both the non‐axenic co‐culture experiments conducted in the present study and in similar experiments with non‐axenic *Tetraselmis* and *Artemia* (Grotkjær *et al*., 2016a). This suggests that the microbiota of the non‐axenic systems have a protective effect on *V. anguillarum;* however, this is not necessarily a problem in an aquaculture context as it is not essential to completely eradicate all vibrios to get a protective effect. Rønneseth *et al*. (2017) found a significant decrease in virulence for several *V. anguillarum* strains tested in turbot, halibut and cod larvae challenge trials when added at low density (10^4^ CFU ml^−1^) as compared to high density (10^6^ CFU ml^−1^). Hence, although *V. anguillarum* was not reduced to below inoculum levels in the presence of *P. inhibens*, our findings support the use of *Phaeobacter* spp. as probiotics in copepod systems. In both co‐culture experiments, *P. inhibens* was able to establish itself at a constant level, which is consistent with its reported association with micro‐ and macroalgae, zooplankton, squids, copepods and fish larvae (Hjelm *et al*., 2004; Rao *et al*., 2005; Venmathi Maran *et al*., 2007; Porsby *et al*., 2008; Collins *et al*., 2012; Gram *et al*., 2015; Grotkjær *et al*., 2016b). This supports the proposal that one way of utilizing *Phaeobacter* spp. in aquaculture is by introducing them directly into the live feed systems (Planas *et al*., 2006; Prol *et al*., 2009; Grotkjær *et al*., 2016b).

Previously, non‐axenic co‐culture experiments have used a chloramphenicol‐resistant *V. anguillarum* as target organism and estimated vibro abundances on solid substrates supplemented with antibiotics (Grotkjær *et al*., 2016a). However, using antibiotic as selection marker is not without challenges. The natural microbiome may harbour bacteria resistant to the antibiotic marker, the antibiotic marker may be unstable, and the marker may affect the fitness of the tagged strain, all of which can result in over‐ or underestimation of the actual abundances (Andersson and Levin, 1999; Allen *et al*., 2010). Several non‐growth dependent methods have been developed for detection and/or quantification of vibrios (Eiler and Bertilsson, 2006; Prol *et al*., 2009; Saulnier *et al*., 2009; Kim and Lee, 2014). However, as previously described, vibrios are part of the established microbiota of live feed, thus genus‐ or species‐specific primers will potentially amplify vibrios from the background microbiota. In this study, we attempted to develop strain‐specific primers for quantification of the pathogenic *V. anguillarum* 90‐11‐286. Three of the 12 tested vibrio strains, including the target strain, were amplified within the limits of detection (*C*_*t*_ ≤ 30). The two non‐target strains were the closely related *V. anguillarum* PF7 and PF4 (Table 2). All three strains are highly virulent and cluster closely when compared by 163 concatenated virulence factors and by core genome phylogeny (Castillo *et al*., 2017; Rønneseth *et al*., 2017). However, the semi‐strain‐specific primers developed provide an effective method for detecting few specific vibrios in non‐axenic systems. As they were made for research and not diagnostic purposes, it is possible to test the applicability of the primer set prior to experimental use.

In conclusion, our study shows that the emerging live feed copepod, *A. tonsa*, potentially can function as an infection vector for pathogenic *V. anguillarum* strains. However, *Vibrio* counts in the copepod cultures were lower than what has previously been seen for *Artemia* and algae cultures, supporting the use of *A. tonsa* as fish larvae feed. *P. inhibens* efficiently inhibited *V. anguillarum* in both non‐axenic copepod and algae cultures supporting its use as a probiotic in live feed systems. Lastly, we have described direct qPCR methodology that provides an effective means of detecting vibrios in complex non‐axenic live feed systems without the use of selection markers, which potentially could affect pathogen behaviour.

## Experimental procedures

### Bacterial strain and culture conditions

Two fish pathogenic *V. anguillarum* strains were used in this study. Strain NB10, used for the adhesion/invasion experiment, was isolated from the Gulf of Bothnia and is pathogenic towards turbot and halibut larvae (Rønneseth *et al*., 2017). The strain has been tagged by insertion of plasmid pNQFlaC4‐gfp27 (*cat*,* gfp*) into the chromosome (Croxatto *et al*., 2007) and was provided by Prof. Debra L. Milton (Department of Molecular Biology, Umeå University). The highly virulent *V. anguillarum* strain 90‐11‐286 (Rønneseth *et al*., 2017) was used in the co‐culture experiments and was isolated from diseased rainbow trout (*Oncorhynchus mykiss*) from a Danish aquaculture (Pedersen and Larsen, 1993; Skov *et al*., 1995). *P. inhibens* DSM 17395 was used for the co‐culture experiments (Martens *et al*., 2006; Vandecandelaere *et al*., 2008; Buddruhs *et al*., 2013). NB10 and 90‐11‐286 were grown and counted on TSA (Tryptone Soy Agar, Difco 212185) supplemented with 2.5 mg l^−1^ chloramphenicol for NB10. The vibrios have very distinct colony morphology on TSA. *Phaeobacter inhibens* DSM 17395 is unable to grow on TSA due to its low salinity, thus TSA functions as a semiselective medium. *Phaeobacter inhibens* was grown and counted on MA (Marine Agare, Difco 2216) where it grows as distinct brown colonies due to Fe‐TDA complex (D'Alvise *et al*., 2016). Bacterial stock cultures were stored at −80°C in 20% (vol/vol) glycerol. Two to three days prior to use, stock cultures were streaked on agar plates and incubated at 25°C. The purity of the bacteria was checked by colony morphology, and single colonies were used for inoculation of each preculture. All bacterial precultures were grown in 20 ml of ½YTSS (2 g Bacto Yeast extract, 1.25 g Bacto Tryptone, 20 g Sigma Sea Salts, 1 L deionized water) (Sobecky *et al*., 1997) at 25°C and 200 rpm for 24 h.

### Preparation of non‐axenic algae and copepods cultures

The non‐axenic *R. salina* K‐1487 originates from Denmark and was provided by Prof. Thomas Kiørboe (National Institute of Aquatic Resources, Technical University of Denmark) (Nielsen and Kiørboe, 2015). A *R. salina* K‐1487 stock culture was maintained in f/2 medium (Guillard and Ryther, 1962; Guillard, 1975) without Na_2_SiO_3_ but with 5 mM NH_4_Cl in 3% IO (Instant Ocean salts, Aquarium Systems Inc.) (f/2‐Si+NH_4_) at 18°C and 24 μmol photons m^−2^ s^−1^, photosynthetically active radiation (PAR). The algal density was determined by counting in a Neubauer‐improved counting chamber, and the culture was adjusted to approx. 100 000 cell ml^−1^ in f/2‐Si+NH_4_ and 25 ml was distributed into 50‐ml falcon tubes. *A. tonsa* eggs were provided by Prof. Benni W. Hansen (Department of Science and Environment, Roskilde University) (Drillet *et al*., 2006; Hansen *et al*., 2010) and were kept at 5°C until use. Two days before the experiment, eggs were inoculated in 3% IO and incubated at 18°C and 24 μmol photons m^−2^ s^−1^, photosynthetically active radiation (PAR). For bacterial mono‐ and co‐culture experiments, 50‐ml falcon tubes were set up with 25 ml 3% IO with 2–3 *Acartia tonsa* nauplii per millilitre. For the invasion experiment, 50‐ml falcon tubes containing 20 ml *R. salina* culture were supplemented with approx. 5 *Acartia tonsa* nauplii per millilitre.

### *A. tonsa* invasion experiment

Precultures of *V. anguillarum* NB10 were 10‐fold serially diluted in 3% IO, and 250 μl dilutions were used to inoculate non‐axenic cultures of *A. tonsa* fed *R. salina*, aiming at an initial concentration of 10^5^ CFU ml^−1^. Experiments were carried out over 96 h where bacterial concentrations and copepod mortality were determined every 24 h. At the end of the experiment, *A. tonsa* from cultures with and without NB10 was investigated for GFP signal using phase‐contrast and fluorescence microscopy at 100× magnification using an Olympus BX51 fluorescence microscope (WIB excitation 460–490 nm, emission >515 nm).

### Co‐culture experiment in non‐axenic *R. salina* and unfed *A. tonsa* cultures

Precultures of *V. anguillarum* 90‐11‐286 were 10‐fold serially diluted in 3% IO, and 250 μl diluted culture was used to inoculate non‐axenic *R. salina* and *A. tonsa* cultures, aiming at an initial concentration of 10^4^ CFU ml^−1^. Precultures of *P. inhibens* DSM 17395 were added undiluted to the non‐axenic *R. salina* and *A. tonsa* cultures, aiming at an initial concentration of 10^7^ CFU ml^−1^. 250 μl ½YTSS was added to uninoculated controls and cultures where only 90‐11‐286 had been added. The cultures were incubated at 18°C, lying horizontally on a rotary shaker at 60 rpm in an algae growth cabinet. Experiments were carried out over 72 h where bacterial and algal abundances, and copepod mortality were determined every 24 h. Samples for DNA extraction for qPCR‐based quantification of 90‐11‐286 were taken every 24 h (see below). Bacterial abundances were determined by CFU counts. 90‐11‐286 counts were determined on TSA, and DSM 17395 counts were determined on MA. Plates were incubated at 25°C. TSA plates were counted after 1 and 2 days and MA plates after 2‐3 days. *R. salina* abundances were counted in a Neubauer‐improved counting chamber. The number of dead *A. tonsa* was determined using a Sedgewick‐Rafter counting chamber. The number of surviving *A. tonsa* was determined after the experiment was terminated. All combinations were made in biological triplicates.

### Primer design and quantification of *V. anguillarum* 90‐11‐286 by qPCR

The primers Fw_90‐11‐286 (5′ ‐ CAACTTAGGCGTGCAATGGG ‐ 3′) and Rev_90‐11‐286 (5′‐ ACCGCTTTACTGGTGGTGG ‐ 3′) were designed using Primer‐BLAST from NCBI (Ye *et al*., 2012). Standard settings were used except for the ‘PCR product size’ which was set to min 75 bp and max 200 bp and the database specifications which was set to Genomes and Bacteria (taxid:2). *V. anguillarum* 90‐11‐286 chromosome I, complete sequence (NZ_CP011460.1) was used as template. For qPCR detection and quantification of the pathogen, genomic DNA was extracted from 1 ml samples using the NucleoSpin Tissue kit (M740952; Macherey‐Nagel, Düren, Germany) as described by the manufacturer. Extracted DNA was stored at ‐20° C until use. Amplification reaction mixtures contained 12.5 μl 2 × SYBR^®^ Green PCR Master Mix (4309155; Applied Biosystems), 1 μl (10 μM) Fw primer, 1 μl (10 μM) Rev primer, 2 μl template DNA and 8.5 μl H_2_O (DNA grade). Reactions were run on a Mx3000P (Stratagene) qPCR System, using the program 1 cycle at 95°C for 10 min, 40 cycles at 95°C for 15 s, 56°C for 1 min and 72°C for 1 min followed by a dissociation curve 95°C for 1 min, 55°C for 30 s and 95°C for 30 s. DNA grade water was included as non‐template controls (NTC), and positive controls consisted of genomic DNA from the target strain. For *in vivo* testing of primer specificity against 11 *V. anguillarum* strains and one *V. harveyi* strain (Table 2), precultures were diluted to 10^7^ CFU ml^−1^ in 3% IO from which TSA plate counts were made and DNA was extracted. For quantification, a standard curve relating *C*_*t*_ (cycle threshold) values to bacterial density (CFU ml^−1^) was made with 90‐11‐286 preculture. The preculture was 10‐fold serial diluted in 3% IO and inoculated into non‐axenic *A. tonsa* cultures aiming for 10^7^ to 10^3^ CFU ml^−1^. *Vibrio* counts of the cultures were made using TSA plates, and DNA was extracted from the samples. The measurements were analysed using GraphPad Prism 7.04 (GraphPad Software, San Diego CA). The limit of detection was estimated based on *C*_*t*_ values of the control samples with only *A. tonsa* and background microbiota.

### Statistical analysis

CFU ml^−1^ values and CFU ml^−1^ of the corresponding *C*_*t*_ values for each biological replicate were log10‐transformed prior to statistical analysis. Statistical analyses of differences were performed using *t*‐test (alpha = 0.05) in GraphPad Prism 7.04 (GraphPad Software, San Diego CA).

## Conflict of interest

The authors declare no conflict of interest.
